# Detection of Multiple Nosocomial *Trichosporon asahii* Transmission Events via Microsatellite Typing Assay, South America

**DOI:** 10.3201/eid3109.241929

**Published:** 2025-09

**Authors:** Elaine C. Francisco, Norma B. Fernández, Mauricio Carbia, Chendo Dieleman, Andra-Cristina Bostanaru-Iliescu, Jos Houbraken, Arnaldo L. Colombo, Ferry Hagen

**Affiliations:** Westerdijk Fungal Biodiversity Institute, Utrecht, the Netherlands (E.C. Francisco, C. Dieleman, J. Houbraken, F. Hagen); Escola Paulista de Medicina-Universidade Federal de São Paulo, São Paulo, Brazil (E.C. Francisco, A.L. Colombo); Hospital de Clínicas José de San Martín, Universidad de Buenos Aires, Buenos Aires, Argentina (N.B. Fernández); Instituto de Higiene, Facultad de Medicina, Universidad de la República, Montevideo, Uruguay (M. Carbia); Iași University of Life Sciences, Iasi, Romania (A.-C. Bostanaru-Iliescu); Antimicrobial Resistance Institute of São Paulo, São Paulo (A.L. Colombo); University Medical Center Utrecht, Utrecht (F. Hagen); Institute for Biodiversity and Ecosystem Dynamics, University of Amsterdam, Amsterdam, the Netherlands (F. Hagen)

**Keywords:** Trichosporonosis, *Trichosporon asahii*, fungi, nosocomial outbreak, pathogen typing, microsatellite typing, antifungal resistance, antimicrobial resistance, emerging opportunistic pathogens, South America

## Abstract

The fungus *Trichosporon asahii* has emerged as a cause of nosocomial infections, particularly in immunocompromised patients. Given its rising prevalence, information on its genetic diversity and transmission dynamics is urgently needed. We developed a microsatellite typing tool to investigate the genetic relatedness of *T. asahii* isolates. We selected 6 microsatellite markers from nanopore long-read sequencing of the *T. asahii* type-strain CBS 2479. We applied those markers to 111 clinical and environmental isolates; microsatellite typing showed high variability among isolates (11–37 alleles per marker) and identified 71 genotypes with strong discriminatory power (Simpson index of 0.9793). We applied the microsatellite typing method to *T. asahii* isolates from South America and identified multiple nosocomial transmission events from hospitals in Brazil, including clusters spanning more than a decade. The panel we developed offers high reproducibility and specificity, making it an effective tool for tracking outbreaks and determining the public health effects of *T. asahii* infections.

*Trichosporon asahii* is an emerging yeast-like fungal pathogen causing life-threatening catheter-related infections worldwide (*1*–*3*). Despite being often overlooked, the occurrence of invasive trichosporonosis has dramatically increased in recent decades, and crude mortality rates have reach up to 80% depending on patients’ underlying conditions (*3*–*6*). Episodes of invasive trichosporonosis caused by *T. asahii* are primarily reported in long-term hospitalized patients with underlying hematologic malignancies and neutropenia, as well as among critically ill patients who have undergone invasive medical procedures, have indwelling medical devices, and have been exposed to broad-spectrum antimicrobial therapy (*3*,*5*). Since the 2000s, cases of invasive trichosporonosis have also been reported in immunocompetent hosts and hospitalized COVID-19 patients, posing new challenges in stratifying at-risk populations (*7*–*11*).

*T. asahii* exhibits a peculiar antifungal susceptibility profile, and is intrinsically resistant to echinocandins, often showing decreased in vitro susceptibility to amphotericin B (*1*,*2*,*12*,*13*), which can exert substantial selective pressure on the growth of this pathogen. Triazoles, particularly voriconazole, are recommended as first-line therapy for treating invasive trichosporonosis (*14*). However, the intraspecific diversity among clinical *T. asahii* isolates can contribute to their reduced susceptibility to triazoles, highlighting the relevance of early diagnosis for effective management of invasive trichosporonosis (*15*,*16*).

Some authors have reported clusters of nosocomial *T. asahii* infections, but epidemiologic typing tools to investigate the potential clonal spread of *T. asahii* in clinical settings are notably lacking (*11*,*17*,*18*). Sequencing of the intergenic spacer (IGS) 1 of the rDNA has been used to explore the intraspecific diversity of *T. asahii*, and 15 IGS1 genotypes have already been described (*19*). However, epidemiologic studies assessing the global distribution of those genotypes have predominantly reported high prevalence rates for IGS genotypes G1–G7. In contrast, other recently identified IGS1 genotypes, G8–G15, have been documented (*15*), underscoring the need for a robust discriminatory typing approach to investigate the intraspecific diversity of clinical *T. asahii* isolates (*15*,*16*,*20*).

Microsatellites, also known as short tandem repeat (STR) units, are widely used as a fast, highly sensitive, and cost-effective typing technique to investigate the molecular diversity within fungal populations during nosocomial outbreaks and for monitoring pathogens over time (*21*). Microsatellite-based typing has been recognized as the optimal tool for population studies and outbreak investigations in healthcare settings, particularly for *Candida auris*, *C. parapsilosis*, *Aspergillus fumigatus*, and *Cryptococcus* spp. (*22*–*27*), providing reliable evidence in the epidemiologic investigations. We developed a microsatellite-based typing tool for *T. asahii* and applied it to a large and genetically diverse collection of clinical and environmental isolates to assess its use in epidemiological investigations.

## Materials and Methods

### Media, Strains, and Standard DNA Extraction

**Figure 1:**
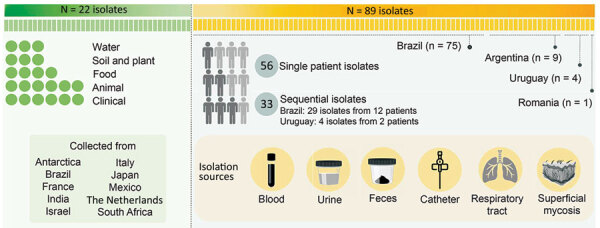
Graphical representation of 111 isolates evaluated in a study of multiple nosocomial *Trichosporon asahii* transmission events, South America. Reference strains are from CBS culture collection (https://wi.knaw.nl/fungal_table) hosted by the Westerdijk Fungal Biodiversity Institute.

To set up the microsatellite typing panel, we used the clinical *T. asahii* type-strain CBS 2479 from the CBS culture collection (https://wi.knaw.nl/fungal_table) hosted by the Westerdijk Fungal Biodiversity Institute (Utrecht, the Netherlands) as a reference and a set of 21 CBS isolates that originated from clinical (n = 5), veterinary (n = 5), and environmental (n = 11) sources. We also used a large set of unique clinical isolates from Brazil (n = 46), Argentina (n = 9), and Romania (n = 1), a set of 29 sequential isolates collected on different days from 12 patients across 6 medical centers in Brazil, and 4 isolates from 2 patients in Uruguay (Figure 1). We cultured isolates onto malt extract agar and incubated for 48 hours at 25°C. We performed DNA extraction by using previously described methods (*28*). We identified all strains as *T. asahii* on the basis of sequencing the IGS1 rDNA locus, as previously described (*15*,*20*).

### Genome Sequencing

The genome of *T. asahii* type-strain CBS 2479 was published a decade ago and was generated at that time by short-read sequencing (National Center for Biotechnology Information [NCBI] BioSample no. SAMN02981437 and BioProject no. PRJNA164647). However, that draft genome was found to be highly fragmented into 78 scaffolds and 342 contigs (*29*). Hence, we resequenced the genome using long-read nanopore sequencing to cover complex genetic regions like the microsatellite loci. We prepared a fresh culture of CBS 2479 onto malt extract agar and incubated for 48 hours at 25°C, then extracted high-quality genomic DNA, as previously described by our team (*30*). In brief, we performed library preparation by using the SQK-LSK109 and EXP-NBD114 Native Barcoding DNA Kits (Oxford Nanopore Technologies, https://nanoporetech.com), following the manufacturer’s instructions. We first loaded the prepared library onto a Flongle flow cell to confirm the quality, then ran the library on a MinION flow cell and base-called raw data by using Guppy version 4.5.4 (all Oxford Nanopore Technologies) using the high-accuracy mode. That process yielded 4.8 Gbp of data with an N50 (sequence length of the shortest contig at 50% of the total assembly length) of 17 kbp and an N90 (sequence length for which the collection of all contigs contains at least 90% of the sum of the lengths of all contigs) of 3.370 bp. We performed de novo genome assembly by using Flye version 2.9 (https://github.com/mikolmogorov/Flye) and the setting –genome-size 24m–min-overlap 10000, which resulted in a genome size of 25,250,028 bp dispersed over 19 fragments with an N50 of 2,305,946 bp, and the largest fragment was 5,662,815 bp. The mean coverage was 198× for the nuclear fragment and 1,310× for the mitochondrial genome that had a length of 31,421 bp after manual curation. We deposited data in NCBI under GenBank accession nos. CP116781–99 for the genome assembly, BioProject accession no. PRJNA926907 for the project description, BioSample accession no. SAMN32886118 for the biological sources, and Sequence Read Archive accession no. SRR23205074 for the sequence reads.

### Development of Microsatellite Typing Panel

We used the fasta-file of the de novo assembled CBS 2479 genome as input for the Tandem Repeat Finder software using the standard parameters (*31*), and included flanking regions for each locus to enable primer design. We detected nearly 4,800 microsatellite loci, which we subjected to the following selection criteria: >10 copies of the repeat unit; >90% of the repeat units were intact, preferentially a dinucleotide, trinucleotide, or tetranucleotide repeat unit; and selected loci were on different fragments of the de novo genome assembly of CBS 2479. That process resulted in an initial list of 26 loci for which we developed primers by using Primer3 version 0.4.0 (*32*) with the standard settings, which we slightly adapted as follows: optimal primer T_m_ 60°C + 1°C, a maximum of 3 poly-X nucleotides, and an optimal primer size of 20 bp (range 18–27 bp). The searched amplicon length was 50–200 bp, excluding the microsatellite loci.

First, we used a set of 8 *T. asahii* isolates, CBS collection nos. CBS 2479, CBS 5599, CBS 7631, CBS 8969; and isolate nos. L2122, L7918, L9206, and L920/2016 from the microorganism bank of the Special Mycology Laboratory at Universidade Federal de São Paulo (São Paulo, Brazil), as the primary test set to determine whether the designed primer sets yielded amplicons for all isolates. We performed PCR in a reaction containing 16.8 µL water, 2.5 µL 10× PCR buffer, 1.0 µL MgCl_2_ (50 mmol), 1.0 µL 0.5 U BIOTAQ Taq polymerase, and 2.5 µL dNTP (1 mmol) (all Bioline Meridian Bioscience, https://www.bioline.com), 0.1 µL 100 pmol/µL unlabeled forward and reverse primer (Integrated DNA Technologies, https://www.idtdna.com), and 1.0 µL DNA template. We performed PCR as follows: initial denaturation at 94°C for 5 minutes, 35 cycles of 94°C for 30 seconds, 60°C for 30 seconds, and 72°C for 1 minute, a final extension for 72°C for 5 minutes, and hold at 21°C. 

To check the success of the designed primer sets, we checked all amplicons by 2% agarose gel electrophoresis. We found 12 of the 26 initial primer sets yielded an amplicon for the set of 8 test isolates; we subsequently tested those 12 primer sets by using a second larger set of 16 additional *T. asahii* isolates, including clinical and environmental isolates from CBS culture collection, using the same PCR procedure. After running the second set, we identified 6 primer combinations that yielded amplicons for all 24 isolates tested and observed size differences by agarose gel electrophoresis. For each of the primer sets, we ordered a primer (Integrated DNA Technologies) with a fluorescein-label to enable detection of amplicons by capillary electrophoresis (Table).

**Table:** Microsatellite typing panel used for detection of multiple nosocomial *Trichosporon asahii* transmission events via microsatellite typing assay, South America*

Accession no.	Contig; reference code†	Expected size; total fragment, bp‡	Repeat unit	*D* value; no. alleles	Forward primer, 5′ → 3′	Reverse primer, 5′ → 3′
CP116786	6; E	173; 206	CA	0.8280; 12	FLU-TCGTCTGTCATCGACCCATA	GGCTCAGCTGAAGCTCACTT
CP116785	5; G	123; 153	GA	0.7731; 11	FLU-TCCCTTTGATTTGGGTGTGT	CTCTCCCAGGTTCGTTTCAA
CP116781	1; I	183; 211	TG	0.7481; 9	FLU-AGCCTTAGTTGCCCTTGTCA	ACTCAACACTTGGGCGACTT
CP116782	2; K	139; 165	AG	0.7666; 10	GATCGAGTCCAAGGAACGAC	FLU-TTCCCGTCCACCTTTACTGA
CP116785	5; P	112; 158	TCGT	0.6600; 14	FLU-ACGAACTCCATGGCTGAGTC	TGACTGACAACACACCCGATA
CP116785	5; Q	135; 169	GTT	0.6452; 12	FLU-ATCTCGGTTGTTGCCGTTAT	GCAACAGCAACAGCAGTACC

*Isolates sourced from CBS culture collection (https://wi.knaw.nl/fungal_table) hosted by the Westerdijk Fungal Biodiversity Institute, Utrecht, the Netherlands. Discriminatory power of microsatellite typing panel assay as determined via the Simpson index of diversity (*D*). FLU, fluorescein. †de novo assembly contig number; microsatellite panel reference code. ‡The expected fragment size without the repeat units; number of alleles refers to the total fragment size of the *T. asahii* type strain CBS 2479.

Finally, we used a large set of 75 clinical isolates obtained from South America and Europe to check for reproducibility, stability, and specificity of the *T. asahii* microsatellite typing panel of 6 loci (Appendix 1 Table). The isolates had been obtained from different anatomic sites, including deep-seated and superficial infections, representing the 5 most prevalent IGS1 genotypes, as previously described (*15*).

For capillary-based fragment analyses, we followed the PCR approach described by performing 6 PCRs using a fluorescein-labeled primer (Table). We checked PCR yields by using 2% agarose gel electrophoresis. Thereafter, we purified amplicons by using Sephadex (Sigma-Aldrich, https://www.sigmaaldrich.com), and arbitrarily diluted amplicons 50–200× with water. We mixed 1 µL diluted amplicon with 10× diluted Orange500 size marker (NimaGen, https://www.nimagen.com) in a 96-well plate, then incubated at 94°C for 1 minute and at 4°C for 1 minute. We obtained raw data by running the fragment analysis on an ABI3700xL Genetic Analyzer (Thermo Fisher Scientific, https://www.thermofisher.com).

### Data Analysis and Discriminatory Power

We analyzed raw data and relatedness between strains by using Bionumerics version 7.6 (Applied Maths, http://www.applied-maths.com) via the unweighted pair group method with arithmetic averages, as previously described (*24*). We determined the discriminatory power of the microsatellite panel by using the Simpson index of diversity (*D*) (*33*). A *D* value of 1 indicates that the typing method was able to discriminate between all isolates, and a value of 0 indicates that all isolates were identical (clonal).

## Results

### Development and Evaluation of *T. asahii* Microsatellite Typing Assay

To develop the typing assay, we selected 6 of 26 promising loci: 4 dinucleotide loci (E, G, I, and K), 1 trinucleotide (locus Q), and 1 tetranucleotide repeat loci (locus P). Loci G, P, and Q were all on the same contig, but loci E, I, and K were on different contigs (Table).

We used a total of 111 *T. asahii* isolates, including 22 CBS reference strains, 56 nonreplicated single-patient isolates, 29 sequential clinical isolates from 12 patients in Brazil, and 4 sequential isolates from 2 patients in Uruguay. The *D* values ranged from 0.6452 for locus Q (tetranucleotide repeat unit) to 0.8280 for locus E (dinucleotide repeat unit) (Table). The combination of all 6 loci yielded a *D* value of 0.9793.

Among the 111 *T. asahii* isolates tested, we identified 71 microsatellite genotypes, in which each genotype contained clusters of 1–11 isolates (Appendix 2 Figure 1). Of the 46 nonreplicated clinical isolates from Brazil, 20 (44%) exhibited unique STR markers. Among the 9 *T. asahii* isolates from Argentina evaluated, 6 displayed unique STR markers, 2 shared identical genotypes, and 1 clustered in a separate group, hitchhiking with isolates collected from both clinical and environmental sources.

**Figure 2:**
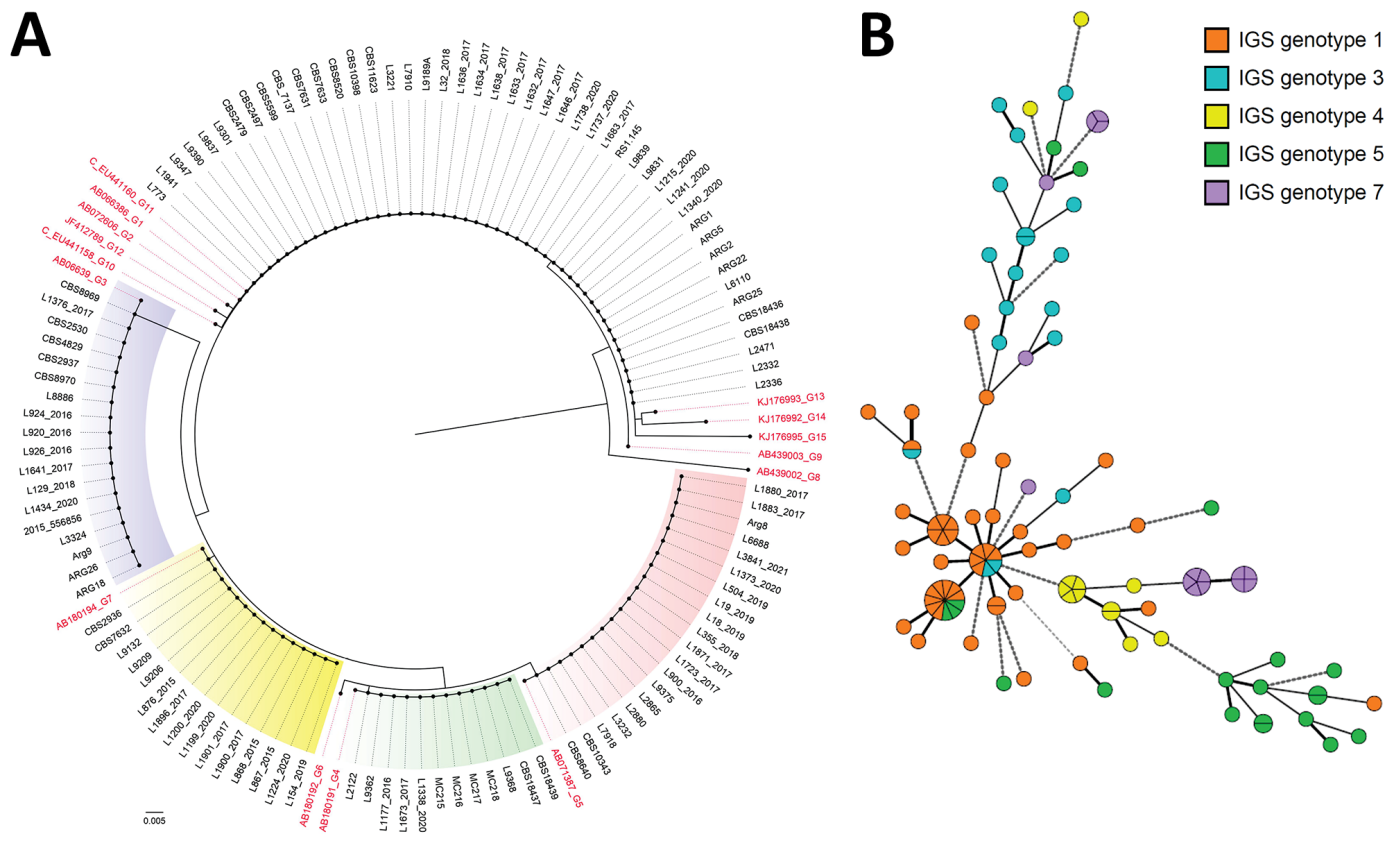
Phylogeny of multiple nosocomial *Trichosporon asahii* transmission events via microsatellite typing assay, South America. We compared IGS1 sequencing–based genotyping and a microsatellite panel for 111 *T. asahii* isolates. A) Neighbor-joining tree based on the rDNA sequencing target, conducted in MEGAX (https://www.megasoftware.net). Red text indicates GenBank accession numbers for reference strains of the described genotypes. Scale bar indicates nucleotide differences per site. B) Minimum spanning tree generated by 6 loci of microsatellite data showing the relationship between microsatellite typing and IGS1 genotypes. Each circle represents a unique genotype; if multiple isolates share an identical genotype, they are shown as a fraction of the circle. Lines between circles indicate the relative similarity, the shorter the line the less prominent the difference between the microsatellite genotypes. Thick lines identify genotypes with fewer differences (1 of 6 loci tested); medium thick lines identify genotypes with differences in 2 of the 6 loci; thick and medium thick dashed lines represent genotypes that share 2–3 identical loci of the 6 tested. Thin dotted lines identify genotypes sharing only 1 identical locus. IGS, intergenic spacer.

Among 12 patients from Brazil who had sequential isolates, 10 exhibited identical or highly related genotypes that differed by <1 marker (Appendix 2 Figure 1). We observed genetically distinct genotypes in the other 2 patients: isolates from patient 8 (1199/2020 and 1200/2020) displayed varying numbers of microsatellite repeat units across all 6 loci examined, and isolates from patient 11 (L1871/2017, 1880/2017, and 1881/2017) differed at loci I and K (Appendix 2 Figure 2). Among the identified clusters, the largest consisted of 11 isolates, 7 of which were from a single hospital in the southwest of Brazil. The first isolate appeared in April 2001 (patient 4), followed by a second in July 2001 (patient 5), and others appeared in July 2014 (patient 6). The second largest cluster included 7 isolates collected from 2 patients at a hospital in the south of Brazil; the first isolate appeared in 2010 (patient 1) and the second in 2015 (patient 2). Of note, we found that 4 sequential isolates from a single hospital in Uruguay, collected from 2 different patients (patient 13, isolates nos. MC215 and MC216; and patient 14, isolate nos. MC217 and MC218), were identical. However, we could not obtain retrospective epidemiologic data for those patients (Appendix 2 Figure 1).

### Comparison of IGS1 Sequence-Based Genotyping and Microsatellite Typing

The neighbor-joining method divided the 111 *T. asahii* isolates into 5 IGS1 genotypes: 47 (42.3%) G1, 17 (15.3%) G3, 12 (10.8%) G4, 20 (18%) G5, and 15 (13.5%) G7. Clinical isolates from Brazil were represented by all 5 of those IGS1 genotypes; 30 (40%) were genotype G1, 16 (21.3%) were G5, 13 (17.3%) were G7, 10 (13.3%) were G3, and 6 (8%) were G4. We observed nonconcordant results between IGS1 sequencing–based genotypes and microsatellite typing, even among isolates sharing identical IGS1 genotypes, such as the 4 G4 clinical isolates from Uruguay (Figure 2; Appendix 2 Figures 1, 2).

### Reproducibility and Specificity of Microsatellite Typing Panel

To test the reproducibility of the developed panel, we independently amplified the first set of 8 isolates >4 times. In all replicate assays, the microsatellite markers showed identical profiles for all isolates evaluated, indicating reproducible results. Finally, to validate the microsatellite typing panel for *T. asahii*, we tested 20 *Trichosporon* isolates representing the 11 different non–*T. asahii* species currently recognized in the genus and added representative isolates of the correlated *Apiotrichum* and *Cutaneotrichosporon* genera. We found the PCR product amplification of the 6 selected loci did not demonstrate the highly species-specific typing toll seen for *T. asahii*.

## Discussion

The *T. asahii* basidiomycete yeast-like pathogen causes a broad spectrum of human infections and has gained increasing prevalence in life-threatening infections worldwide (*3*,*5*,*12*,*14*). *T. asahii* is recognized as the second or third non–*Candida-*related yeast detected in invasive infection episodes and competes with *Cryptococcus* as leading basidiomycetous yeast pathogen (*3*,*14*). IGS1 sequence-based genotyping is the preferred approach to investigate genetic diversity among *T. asahii* isolates (*3*,*15*,*34*). Despite its relevance, the IGS1-based genotyping can lack the genetic diversity required for outbreak and epidemiologic typing tools, making it difficult to apply in nosocomial outbreak investigations (*8*,*35*). We developed a microsatellite typing panel to genotype *T. asahii*, which can be a valuable complement to IGS1 sequencing genotyping.

Microsatellites, short repetitive DNA sequences, are widely used in molecular studies to explore the genetic relatedness between isolates within fungal populations. This typing tool has greatly contributed to the advancement of epidemiologic typing for a variety of human pathogens, including *C. auris*, *C. parapsilosis*, *Nakaseomyces glabratus* (syn. *C. glabrata*), *Cryptococcus neoformans*, *Cryptococcus deneoformans*, *Cryptococcus deuterogattii*, and *Aspergillus* spp. (*25*–*27*,*34*,*36*–*40*). Compared with other DNA-typing tools, such as internal transcribed spacer–based and amplified fragment length polymorphism (AFLP) profiling, microsatellites have demonstrated superior performance in epidemiologic studies (*23*,*24*,*41*,*42*).

The *T. asahii* microsatellite typing panel we describe consists of 6 loci and revealed remarkable genetic diversity among *T. asahii* isolates. This novel panel successfully distinguished 78 unique isolates (comprising 22 CBS reference isolates and 56 clinical isolates from single patients) into 58 distinct genotypes, a substantially better genetic discriminatory power than the IGS1 sequencing genotyping method, which identified only 5 IGS1 genotypes in the same isolate set. Of note, our panel effectively differentiated between isolates on the basis of their origins, anatomic sites, and year of isolation. Moreover, similar to IGS1 sequencing genotyping, we observed 100% similarity among sequential isolates obtained from 9 of the 12 patients from Brazil from whom multiple isolates were available. Sequential isolates from the 3 other patients were distributed across different microsatellite genotypes; all those sequential isolates were collected 2–7 days after the first isolate, suggesting potential coinfection of those patients by different *T. asahii* genotypes. To underscore the limitation of IGS1 genotyping, all those sequential isolates shared the same IGS1 genotype.

Most isolates we investigated clustered with their sequential counterparts on the basis of microsatellite profiles, suggesting co-infection or cocolonization by the same strain. When we applied our microsatellite assay, we uncovered several clusters spanning single or multiple hospitals. Because microsatellite typing assays are known for high resolution (*27*), the isolates with identical genotypes suggest nosocomial transmission. In the Brazil healthcare setting, the application of this microsatellite panel could provide an accessible and effective strategy to monitor the spread and evolution of *T. asahii* infections. The panel proved particularly useful in different hospital contexts, as demonstrated in 1 outbreak spanning 13 years in a hospital in the southeastern region and another outbreak cluster of 2 patients from southern Brazil. This panel offers a high-resolution intermediate solution for low- and middle-income countries and can be a valuable tool to complement IGS1 sequencing, whereas next-generation sequencing (NGS) remains costly and is not yet widely accessible. In addition, the 4 identical isolates from Uruguay suggest potential interhospital transmission. 

Although the described microsatellite genotyping tool might offer a high-resolution intermediate, future studies should seek further validation of this panel in other hospitals and countries with different epidemiologic profiles. To date, no prior nosocomial *Trichosporon* transmission has been reported, which could indicate a rare event but could also be the result of a lack of genotyping investigations. Previously, potential genetic relationships among *T. asahii* clinical isolates were assessed using a multilocus sequence typing (MLST) tool, but that approach revealed a relatively low level of genetic diversity (*11*,*35*). This study highlights multiple events of nosocomial *T. asahii* transmission in hospitals in South America.

Few studies comparing different typing tools for *T. asahii* are available. IGS1 sequencing is a powerful tool and is considered the standard to discriminate the Trichosporonales genera and species in reference laboratories globally, even for the different genetic lineages within *T. asahii* (*20*). A 2019 study used ISG1 sequencing for molecular characterization of a set of locally collected *Trichosporon* species isolates in Brazil (*2*). That endeavor led to identification of a novel genetic lineage, *T. austroamericanum*, in 2024 (*43*). *T. asahii* IGS1 genotypes G1, G3, G5, and G7 have been extensively associated with *Trichosporon* spp. infections worldwide (*15*,*16*,*19*,*20*,*44**–**46*). However, the various epidemiologic studies demonstrate IGS1 sequencing genotyping lacks the resolution needed for outbreak typing (*15*,*16*,*20*,*44*,*45*,*47*). In this study, we considered isolates from those 5 genotypes at all stages of the research, highlighting their potential application in diverse clinical scenarios.

In a study of the genetic relationship between *T. asahii* isolates from elderly patients hospitalized in a single care center in Spain (*48*), one group supplemented IGS1 sequencing with the since-discontinued commercial DiversiLab typing tool (bioMérieux, https://www.biomerieux.com). However, the addition of that repetitive element PCR typing tool led to inconclusive results because the fingerprint patterns lacked sufficient discriminatory power. The genomewide-based AFLP analysis typing tool has been shown to be an informative approach for fungal outbreak investigations but has been reported only once for *Trichosporon* (*49*). Unfortunately, the selective primer combination used in that study resulted in AFLP profiles that could not distinguish potential related isolates from the unrelated ones (*49*). Moreover, AFLP genotyping has been found to be more laborious, more costly, and less reproducible than other methods, such as microsatellite typing. Hence, microsatellite typing has gradually replaced AFLP to investigate outbreaks caused by fungal pathogens.

A 2023 study published an MLST assay consisting of sequencing from 5 nuclear loci applied to a set of 51 clinical *T. asahii* isolates from Thailand (*35*). The authors of that study concluded that their MLST was useful for population structure analysis, but it seemed to have limited genetic diversity for use in outbreak investigations because the 51 isolates were dispersed among only 5 sequence types (*35*). Another study used whole-genome sequencing (WGS) on a subset of 32 of 54 *T. asahii* isolates that were collected over ≈17 years (*46*). Initially, those isolates were typed using IGS1 sequencing; thus, the finding that short-read genome sequencing resulted in a higher discriminatory power than the former IGS1 typing approach is not surprising (*46*). Because of its high resolution, WGS could soon be used for investigating fungal nosocomial outbreaks. However, WGS is currently costly and technically demanding, making it inaccessible for many diagnostic laboratories in low- and middle-income countries (*50*). In contrast, microsatellite typing offers a more affordable, accessible, and technically simpler alternative, providing sufficient resolution for outbreak investigation at a fraction of the cost of WGS (*36*,*38*). The microsatellite approach we describe provides a middle ground, enabling effective genetic typing of *T. asahii* strains in resource-limited settings. In addition, this method can complement IGS1-based genotyping (*50*) and can be widely implemented in countries without the extensive infrastructure required for WGS.

In conclusion, we assessed intrahospital *T. asahii* transmission by using microsatellite typing, which suggested multiple events of nosocomial transmission by this pathogen in hospitals in South America. The panel we developed offers high reproducibility and specificity, positioning it as an effective epidemiologic tool for tracking *T. asahii* outbreaks and understanding the public health effects of *T. asahii* infections. Our findings highlight the need for ongoing surveillance and effective control measures in hospital settings to mitigate the public health threat of this emerging fungal pathogen.

Appendix 1 Sequence data used for detection of multiple nosocomial *Trichosporon asahii* transmission events via microsatellite typing assay, South America.

Appendix 2 Additional information on detection of multiple nosocomial *Trichosporon asahii* transmission events via microsatellite typing assay, South America.
