# Supplementary material for: Detection of Multiple Nosocomial Trichosporon asahii Transmission Events via Microsatellite Typing Assay, South America
Source: Emerg Infect Dis. 2025 Sep;31(9):1774–82. doi: 10.3201/eid3109.241929 (PMC12407203; doi:10.3201/eid3109.241929)
Supplement: Appendix 2 — Additional information on detection of multiple nosocomial Trichosporon asahii transmission events via microsatellite typing assay, South America. [file 24-1929-Techapp-s2.pdf]

Article DOI: https://doi.org/10.3201/eid3109.241929

EID cannot ensure accessibility for supplementary materials supplied by authors. Readers who have difficulty accessing supplementary content should contact the authors for assistance.

Detection of Multiple Nosocomial *Trichosporon asahii* Transmission Events via Microsatellite Typing Assay, South America

Appendix 2

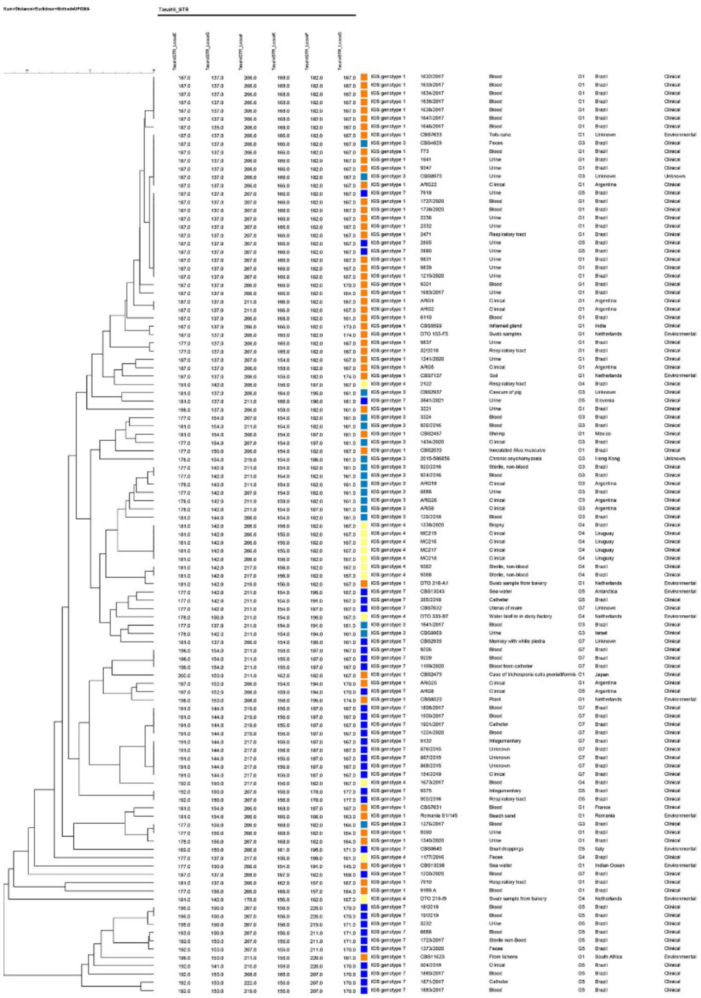

Appendix 2 Figure 1. UPGMA dendrogram showing genotypic diversity among 111 *Trichosporon asahii* isolates obtained from clinical and environmental samples. The scale bar indicates the percentage

similarity between the genotypes. The dendrogram was generated based on analysis of six microsatellite markers. The colors represent the five IGS1 genotypes identified in this study: orange for G1, light blue for G3, yellow for G4, green for G5, and purple for G7. Isolates from the same patient are denoted with the same coding. Clinical isolates labeled as ‘unknown’ refer to isolates of human origin where the specimen source was not documented.

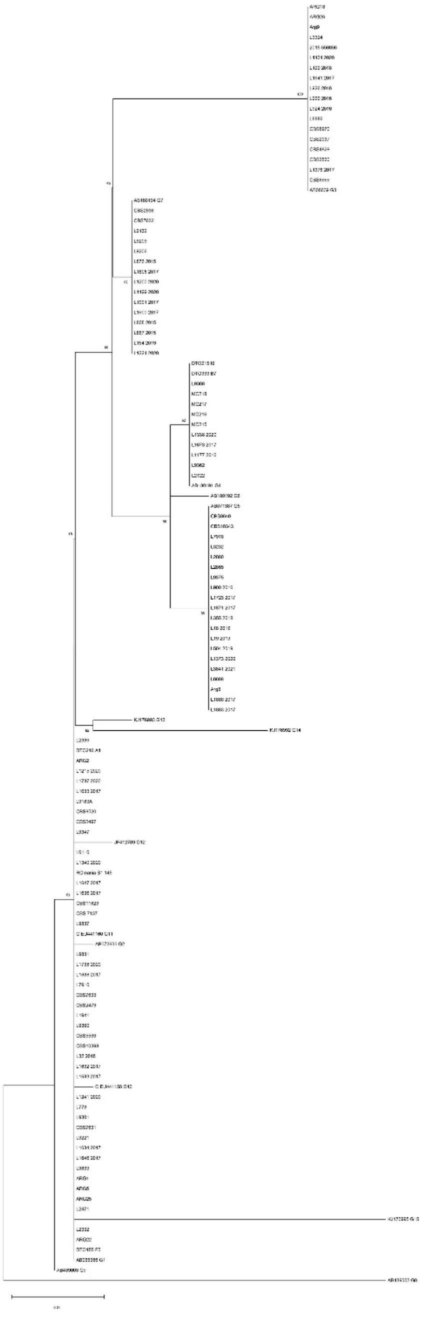

Appendix 2 Figure 2. The phylogenetic analyses of 111 *T. asahii* isolates and the IGS1 genotypes was inferred using the neighbor-joining method. The optimal tree is shown. The percentage of replicate trees in which the associated taxa clustered together in the bootstrap test (1,000 replicates) are shown next to

the branches. The tree is drawn to scale, with branch lengths in the same units as those of the evolutionary distances used to infer the phylogenetic tree. The evolutionary distances were computed using the Kimura 2-parameter method and are in the units of the number of base substitutions per site. This analysis involved 126 nt sequences, conducted in MEGA X.
